# Monthly Summary

**Published:** 1894-11

**Authors:** 


### Monthly Summary.

NECROSIS, CAUSED BY THE NON-ERUPTION OF A DECIDUOUS MOLAR.—By J, R. Megraw, D. D. S., Fayette, Mo.—On May 21st, a physician, aged twenty-four years, asked advice concerning a discharge of pus from the gums surrounding the second upper bicuspid. The bicuspids and molars were sound, but directly over the root of the second bicuspid there was a slight swelling and a small opening, from which pus was exuding. The discharge had existed for five years, with a dull pain at times lasting from a few minutes to several hours. Probing about an inch, I came in contact with a rough substance which could be moved around about a fourth of an inch. I applied a 20 per cent. solution of cocain to the gums, and with a lance made a transverse incision of the gums about an inch long, dissecting it away from the process.

After checking the bleeding, I discovered a molar crown lying length ways, with the top pointing backward. With forceps I removed the tooth and also several small pieces of bone. With a large rose head bur and a rough plug finishing file I thoroughly scraped the bone, syringing the cavity with a solution of listerin, phenol sodique and water, equal parts, then packed the cavity with a 5 per cent. iodoform, with directions to change every two days. The cure was complete. —*Items of Interest.*

PROMISCUOUS EXTRACTION OF TEETH.—At the last meeting of the Alabama State Dental Society the subject of the promiscuous extraction of teeth by irresponsible individuals, was brought before the Society in the President's Address. The subject elicited a lively discussion from several able talkers, and the result was that a committee was appointed to have the law so amended as to prohibit the extraction of teeth "for fee or reward," except by those who have obtained license from the Board of Examiners. There is not the shadow of a doubt that thousands of good, useful teeth are sacrificed every year by these traveling fakirs and painless extractors. We know it is an outrageous and inhuman practice which should not be tolerated; but how are we to reach them? The amendment that Alabama asked for will not reach them. The words for "fee or reward" is the loop-hole through which they slip. Not one of these patent medicine fakirs ever *charge* for extraction. They extract the teeth without pain and without charge as an advertisement for their secret nostrums, which they sell. It is almost impossible to legislate against it. No legislature will pass a law prohibiting the extraction of teeth in cases of emergency "without fee or reward," and we do not believe it would be a humane act to do so. Many people in thinly settled places are inaccessible to a licensed dentist, and many cases of emergency arise in such districts that are quickly relieved by some neighbor who possesses a pair of forceps. It would not be right to pass a law fixing a penalty for extracting teeth in these emergency cases, and yet if we except these cases it leaves a loop-hole for the fakir to get in his dirty work.—*Southern Dental Journal.*

---

TREATMENT OF ROOT CANALS.—Dr. Schrier, of Vienna, uses a preparation of metallic potassium and iodium for removing the putrescent particles out of root canals and for cleansing them. He bases his theory on the saponifying of the contents of the canals and thereby easily removed with warm water. He also considers it a good antiseptic treatment of the roots, and shortens the time considerably for treatment and filling.

CARIES OF THE ALVEOLUS.—As a general rule, dentists have not in the past given as much attention to pathological studies as they should. Our best men have been ambitious to shine as operators, or as mechanics, and have neglected the first and most important of all studies, if we are to be considered in any sense as medical men. Beautiful fillings are too often inserted in teeth that are not in a physiological state, or which are in relation with diseased tissues, and the consequences are sometimes very serious. Many teeth are extracted simply because an otherwise excellent operator is not skilled in diagnosis and treatment. Diseases which, if properly treated at the outset, might be easily cured, are not promptly recognized, and are temporized with, receiving only simple topical applications, till the general surgeon must be called in to remedy the effects of the lack of knowledge. Serious tumors have been dallied with till they have invaded tissues which should have been saved from their ravages.

Dr. W. W. Kinkead, of Nashville, Tenn., in the *Columbus Medical Journal*, gives the following example of a reliable treatment for an acute cold, and for the incipient stage of inflammations of the air passages, as tonsillitis, bronchitis, etc.

| | | |
|---|---|---|
| R | Atropin sulfatis, - - | gr. 1-60-1-100 |
| | Morphin sulfatis, - | gr. ss. |
| | Water, - - - | f ℥ ij. |

M. Sig.: One teaspoonful every half hour.

—*Items of Interest.*

---

ARISTOL dissolved in campho-phenique is a "dead shot" on abscesses, and mixes well with chloro-percha for filling root-canals. I do not doubt that a weak solution of trichloracetic acid with pumice is more effective for cleaning teeth than tincture of iodin, but what is the objection to a weak solution of aromatic sulphuric acid used that way, finishing with a pleasant saponaceous tooth-powder? I have done so for twelve years, and, if not mistaken, have to thank Dr. T. B. Welch for the idea.—C. H. Thorn in *Items of Interest.*

